# Comparative effectiveness of a complex Ayurvedic treatment and conventional standard care in osteoarthritis of the knee – study protocol for a randomized controlled trial

**DOI:** 10.1186/1745-6215-14-149

**Published:** 2013-05-23

**Authors:** Claudia M Witt, Andreas Michalsen, Stephanie Roll, Antonio Morandi, Shivnarain Gupta, Mark Rosenberg, Ludwig Kronpaß, Elmar Stapelfeldt, Syed Hissar, Matthias Müller, Christian Kessler

**Affiliations:** 1 Institute for Social Medicine Epidemiology and Health Economics, Charité -Universitätsmedizin Berlin, Berlin, Germany; 2 University of Maryland School of Medicine, Center for Integrative Medicine, Baltimore, USA; 3 Department for Complementary Medicine, Immanuel Hospital, Berlin, Germany; 4 Ayurvedic Point, School of Ayurvedic Medicine, Milan, Italy; 5 Department of Kaya Cikitsa, JS Ayurveda College and PD Patel Ayurveda Hospital, Nadiad, India; 6 European Academy for Ayurveda, Birstein, Germany; 7 Department of Gynecology and Obstetrics, Hospital of Rotthalmünster, Rotthalmünster, Germany; 8 Central Council for Research in Ayurvedic Sciences (CCRAS), New Delhi, India; 9 Department for Surgery, Immanuel Hospital, Berlin, Germany

**Keywords:** Ayurveda, Ayurvedic, Comparative-effectiveness research, Whole medical system, Osteoarthritis of the knee, Randomized trial, Traditional Indian medicine

## Abstract

**Background:**

Traditional Indian Ayurvedic medicine uses complex treatment approaches, including manual therapies, lifestyle and nutritional advice, dietary supplements, medication, yoga, and purification techniques. Ayurvedic strategies are often used to treat osteoarthritis (OA) of the knee; however, no systematic data are available on their effectiveness in comparison with standard care. The aim of this study is to evaluate the effectiveness of complex Ayurvedic treatment in comparison with conventional methods of treating OA symptoms in patients with knee osteoarthritis.

**Methods and design:**

In a prospective, multicenter, randomized controlled trial, 150 patients between 40 and 70 years, diagnosed with osteoarthritis of the knee, following American College of Rheumatology criteria and an average pain intensity of ≥40 mm on a 100 mm visual analog scale in the affected knee at baseline will be randomized into two groups. In the Ayurveda group, treatment will include tailored combinations of manual treatments, massages, dietary and lifestyle advice, consideration of selected foods, nutritional supplements, yoga posture advice, and knee massage. Patients in the conventional group will receive self-care advice, pain medication, weight-loss advice (if overweight), and physiotherapy following current international guidelines. Both groups will receive 15 treatment sessions over 12 weeks. Outcomes will be evaluated after 6 and 12 weeks and 6 and 12 months. The primary endpoint is a change in the score on the Western Ontario and McMaster University Osteoarthritis Index (WOMAC) after 12 weeks. Secondary outcome measurements will use WOMAC subscales, a pain disability index, a visual analog scale for pain and sleep quality, a pain experience scale, a quality-of-life index, a profile of mood states, and Likert scales for patient satisfaction, patient diaries, and safety. Using an adapted PRECIS scale, the trial was identified as lying mainly in the middle of the efficacy-effectiveness continuum.

**Discussion:**

This trial is the first to compare the effectiveness of a complex Ayurvedic intervention with a complex conventional intervention in a Western medical setting in patients with knee osteoarthritis. During the trial design, aspects of efficacy and effectiveness were discussed. The resulting design is a compromise between rigor and pragmatism.

**Trial registration:**

NCT01225133

## Background

Among chronic diseases, osteoarthritis (OA) is becoming increasingly significant and is responsible for a major part of the disease burden, work disability, and healthcare costs in Germany, Europe, and worldwide [1]. The guidelines for treatment of OA of the knee from the National Institute for Health and Clinical Excellence, the American College of Rheumatology and the European League Against Rheumatism recommend nondrug treatments, including the education of patients, social support, physical exercise, and weight loss [2-5]. Nonsteroidal anti-inflammatory drugs (NSAIDs) are still used as the initial treatment in primary care [6,7]; however, they are associated with a number of side effects, such as upper gastrointestinal bleeding and renal failure [8], as well as myocardial infarction and stroke, especially in the COX-2 inhibitor category [9]. Patients with osteoarthritis as *pars pro toto* for chronic diseases often seek complementary and alternative medicine (CAM) therapies [10].

Ayurveda, is the most prominent medical system of traditional Indian medicine, and is commonly used throughout South Asia. It has been practiced there as a whole system of medicine for more than 2000 years. Ayurveda is one of the oldest systems of medicine worldwide and is acknowledged as a medical science by the World Health Organization [11-13]. In India alone, more than 400,000 Ayurvedic physicians are officially registered; Ayurveda can be studied and applied systematically at more than 250 government-accredited universities or colleges [14]. Ayurveda is also playing an increasing role in European and North America, since its broad introduction in Western countries in the 1980s. At present, it is one of the fastest-growing CAM therapies worldwide [12,15-22]. Ayurveda claims to be effective in treating chronic diseases of the musculoskeletal system [23-25]. It uses complex and individually tailored interventions, including manual therapies, lifestyle and nutritional advice, dietary supplements, medication, yoga, and purification measures [25].

Ayurveda has its own sophisticated diagnostic system; OA generally belongs to a cluster of diseases in which the Ayurvedic principle of kinetic energy, ‘vata’, prevails. Thus, a reduction and regulation of the aggravated principle of kinetic force stands to the fore of a complex Ayurvedic treatment approach for OA of the knee [26]. However, the conventional diagnosis ‘osteoarthritis of the knee’ cannot be directly translated into the Ayurvedic diagnostic system. As an approximation, the Ayurvedic term ‘[janu-] sandhi-gata-vata’ (literal translation from Sanskrit: ‘vata is seated [has moved] in [into] the [knee-] joint’) is most commonly used by the Ayurvedic fraternity. However, sometimes other Ayurvedic diagnoses may also apply (for example, khuda-vata, ama-vata, jirna-vata, vata-rakta). According to Ayurveda, the causes of OA are most often attributed to improper diet, unfavorable life style, trauma, aging processes, and constitutional predispositions. This favors an aggravation of the principle of vata, responsible for all movement, musculoskeletal, and locomotor functions in the body. The aggravated principle of vata brings dryness (rukshata), lightness (laghutva), porosity (saushirya), and coarseness (kharatva) into the joints. Corresponding to Ayurvedic models of pathogenesis, the disease is caused when the aggravated principle of vata settles in the knee joint and begins to destroy the structure and function of the joint. The features seen in OA and sandhi-gata-vata are similar. In the Ayurvedic disease-entity, pain in the knee joint (sandhi-shula) is the main feature and can be accompanied by other features including swelling (shotha), stiffness (stabdhata), crepitus (atopa) and difficulties in performing proper functions of the knee joint [23,24,27-29].

Most noticeably, Ayurveda and conventional Western medicine are based on different sets of logical axioms. It can be difficult to identify precise correspondences between related disease entities within these two systems of disease classification [30-35]. Mean-value based medical strategies are avoided in the constitution-based Ayurvedic approach. Moreover, nomenclatures for disease entities are seen to be of lower importance than nomenclatures for ‘milieu interior changes’ in Ayurvedic medicine [25].

Besides symptom detection, Ayurvedic diagnosis involves a general investigation into a broad spectrum of internal and external conditions, including physiological, metabolic, kinetic, excretory and mental functions, life style, food habits, social and other factors, all capable of developing disharmonies within the patient’s mind-body continuum. By inquiring into a patient’s history and investigations based on refined sense perception (palpation, auscultation, percussion, inspection, and so on), these factors are analyzed and evaluated. Pathogenic disharmonies are classified in terms of dynamic Ayurvedic principles, which cannot be directly equated with modern entities (and have to be explained in other ways, for example, dosha, agni, srotas, dhatu, mala, ama). These principles are found in distinct states and individual pathological constellations and may result in specific symptoms. Furthermore, healthy states and disease are seen as a continuum in Ayurveda. Diagnosis is believed to be the definition of a snapshot within a constant flow of physiological and pathophysiological factors. This enables the Ayurvedic physician to treat a case without giving the disease a name, by dealing with the aforementioned principles [23].

As an example, osteoarthritis of the knee may be accompanied by (in Ayurveda) so-called ‘aggressive intermediate metabolic products’ (ama), leading to systemic or local joint pathologies, resembling, but not equating, features of rheumatoid arthritis, other autoimmune conditions or gout. Consequently, the Ayurvedic diagnosis may not be (janu-) sandhi-gata-vata, but another diagnosis, such as ama-derived situations (ama-vata) or the effects of a ‘hot-acidic blood tissue’ (vata-rakta). However, in conventional medicine, the diagnosis osteoarthritis of the knee would remain the same in all three cases. Ultimately, an experienced Ayurvedic physician would treat the principles of vata, ama, or rakta, rather than a circumscribed diagnosis. Summarized, standardized diagnosis and therapy are avoided in constitutional Ayurvedic medicine; the overall situation of the patient is analyzed, rather than merely the disease [25].

There is some clinical evidence of the effects of Ayurvedic therapies for OA that derives from a limited amount of clinical studies, largely from South Asia [26,36-70]. Most of these trials show limitations regarding their trial designs or methodological transparency. However, Ayurveda is a whole medical system using complex treatment approaches. In theory and practice, it is fundamentally based on constitutional axioms. Ayurveda (alongside its somatic approaches) intentionally includes mind-body and psychosomatic aspects in diagnosis and therapy of all diseases. Complex treatments follow the theory that the combination of different treatment elements exerts synergistic effects and is relevant for the outcome [71,72]. However, to date, no clinical study on OA has been performed that has taken into account the multidimensional approach of Ayurveda as a complex and whole medical system. Another limitation of all previous randomized controlled studies on Ayurveda is that they have focused only on structural Western diagnoses and disease cognitions without considering the fundamental principles of the traditional Ayurvedic diagnostic approach.

As for Western countries, there are very limited amounts of clinical data on single Ayurvedic interventions (for example, leeches [73,74] and ginger extract [75,76]) and no data for complex Ayurvedic treatment of OA. To date it is unknown whether such a complex treatment approach has a clinically relevant effect and whether its effectiveness can exceed the effectiveness of conventional standard care for OA. Moreover, it remains unclear whether local and geographical aspects influence the therapeutic outcome, for example, when Ayurveda is being practiced outside its countries of origin. Overall, there is a need for more evidence from comparative studies in medicine [77], in order to assist healthcare stakeholders in informed decision making. This is also the case for traditional Indian medicine as *pars pro toto* for CAM and is especially recommended for the comparison of whole medical systems like Ayurveda [78]. The generation and synthesis of evidence comparing the benefit and harm of alternative methods to prevent, diagnose, treat, and monitor a clinical condition or to improve the delivery of care is called comparative-effectiveness research [79].

Our clinical hypothesis is that 12 weeks of complex Ayurvedic treatment based on Ayurvedic diagnosis is more effective than 12 weeks of conventional standard care in the treatment of OA of the knee (based on the WOMAC Index). The aim of this study is to compare and evaluate the effectiveness (measured with the WOMAC Index) of a complex individualized Ayurvedic treatment based on Ayurvedic diagnosis in comparison with conventional medical care for osteoarthritis of the knee following current international treatment guidelines for patients with OA of the knee.

## Methods

### Patients

We include patients who fulfill the following criteria: men and women aged 40 to 70 years, who have OA of the knee, as prediagnosed by a specialist (such as an orthopedic specialist or surgeon or a radiologist) according to the American College of Rheumatology criteria [3,80]. Radiologic changes of the knee in the images used by the specialist when diagnosing OA should be a Kellgren-Lawrence score of at least grade 2 in X-ray [81,82] or a Recht grading score of at least grade 2(a) in MRI [83-85]. The patients will have reported a mean pain intensity in the affected knee of ≥40 mm on a 100 mm visual analog scale over the 7 days before baseline assessment, and provide written informed consent.

Patients are excluded for the following criteria: the pain in the knee is caused by congenital dysplasia of the affected knee, rheumatoid arthritis, autoimmune diseases, malignancies, knee surgery or knee-arthroscopy; the patient has been administered any chondroprotective drugs, intra-articular injection into the affected knee joint or systemic medication with corticosteroids during the preceding 3 months; the patient has begun any new treatment for OA during the previous 4 weeks (except analgesic treatment with paracetamol or NSAIDs available over-the-counter); the patient is pregnant or breastfeeding; the patient has an acute mental disorder, a serious acute organic disease, or a serious chronic comorbidity, or is obese (at least WHO grade II); the patient has a blood coagulation disorder, or takes coagulation-inhibiting medication other than acetylsalicylic acid and clopidogrel; invasive measures have been performed at the affected joint during the previous 12 weeks or are planned for the following 12 month; or the patient is in the process of applying for pension or disability benefits. Furthermore, patients are excluded if they had any serious comorbidity that made it impossible to participate in the trial interventions (for example, heart failure NYHA IV).

Patients are recruited from the Immanuel Hospital and Charité Medical University Berlin outpatient clinics. In addition, patients are being informed about the trial in media reports and advertisement in local newspapers. The study was approved by the ethics committee of the Charité University Medical Center (No. EA1/124/10) and is conducted according to the common guidelines for clinical trials (Declaration of Helsinki). The trial registration number is NCT01225133. Informed consent is obtained for all patients. The trial is in the recruitment phase.

### Randomization

Patients are randomized to Ayurvedic or conventional treatment in a 1:1 ratio on the basis of a stratified (strata defined by study site) block-randomization (variable block lengths). The study biometrician generated a randomization list with SAS/STAT software (version 9.1, SAS Inc., Cary, NC). The randomization list is stored by the data manager on a secure database (Microsoft Office Access 2007), where the randomization list is not accessible to any other staff members or study physicians. After registration of the patient details in the secure database, the study nurse has to click a button to perform randomization. Each patient can be only registered and randomized once: the database does not allow any patients details to be deleted.

### Study design

**Figure 1:**
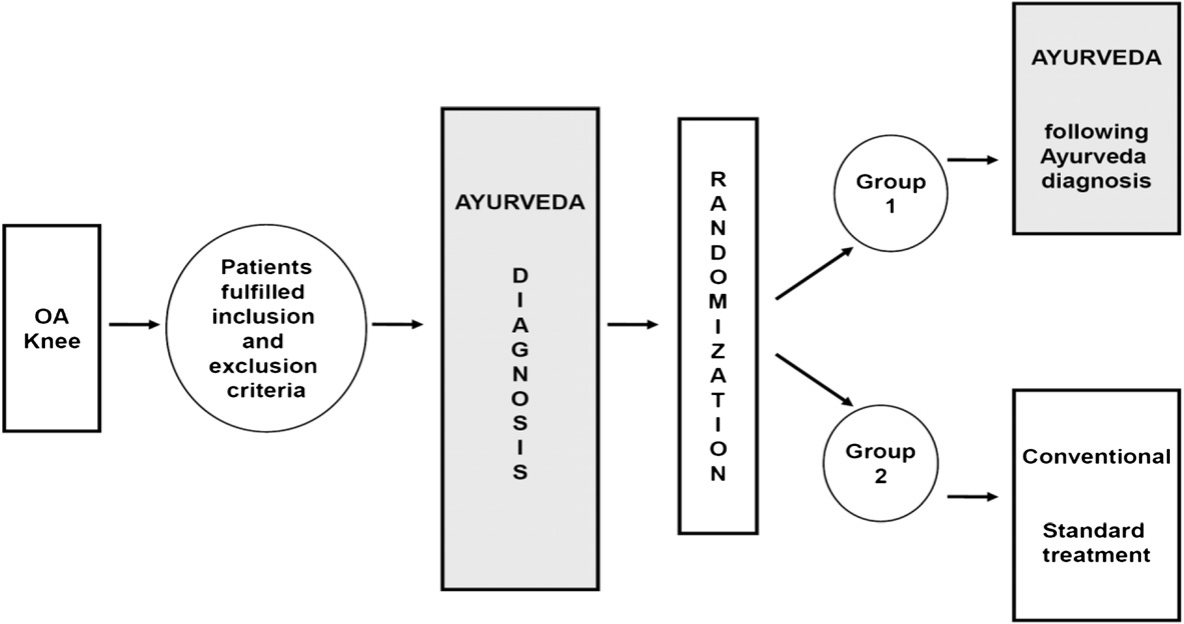
Trial design.

In this prospective, multicenter, randomized controlled trial, all patients included into the study receive an Ayurvedic diagnosis before randomization. After randomization, patients in the Ayurveda group receive a complex individualized Ayurvedic treatment tailored according to their Ayurvedic diagnosis. Patients randomized into the conventional treatment group will receive conventional medical treatment following current guidelines (Figure 1). Because all patients receive the Ayurveda diagnosis, we exclude the context effects of this procedure from our results (for further explanations on design aspects see [86]). The treatment will be performed in two trial centers in Berlin, Germany.

To improve the validity of the Ayurvedic diagnosis within the trial, the first 30 patients recruited for the trial were included in an embedded diagnostic trial (to be reported elsewhere). After inclusion into the trial, they were diagnosed by four independent Ayurvedic medical specialists, each of whom documented an individualized treatment plan for the patient with the help of a standardized form. All assessment and treatment plans for the first 30 patients were discussed between the doctors until consensus was reached for each patient. The results will be reported elsewhere.

### Outcomes

The primary outcome measure will be rated using the Western Ontario and McMaster University Osteoarthritis Index (WOMAC) after 12 weeks [87,88]. We used the 10-point numeric rating scale, as described in the German version, and normalized the results to a 100-point scale. If the patient has bilateral OA of the knee and both knees fulfill the eligibility criteria, the WOMAC Index is used for the same knee throughout the whole study period; the knee that affects the patient most at baseline. Secondary outcome measures will be made using WOMAC subscales (pain, function and stiffness), a pain disability index, a visual analog scale for pain and quality of sleep (0 to 100 mm), a pain experience scale, a quality-of-life index, a profile of mood states, Likert scales for general health-related patient satisfaction, and a patient diary for interventions and co-interventions (medication, yoga or exercise, self-applied massage, health services usage), and safety records (adverse events, serious adverse events). Outcomes will be documented at baseline, at 6 and 12 weeks, and at 6 and 12 months.

### Interventions

#### Ayurveda group

The Ayurveda intervention is multimodal and follows the principles of Ayurveda as a whole medical system. The Ayurveda intervention was developed over 18 months in an international consensus procedure including experienced Ayurveda experts from India, Germany, and Italy. In addition, it was based on classical Ayurvedic literature [23,24,27,28].

**Figure 2:**
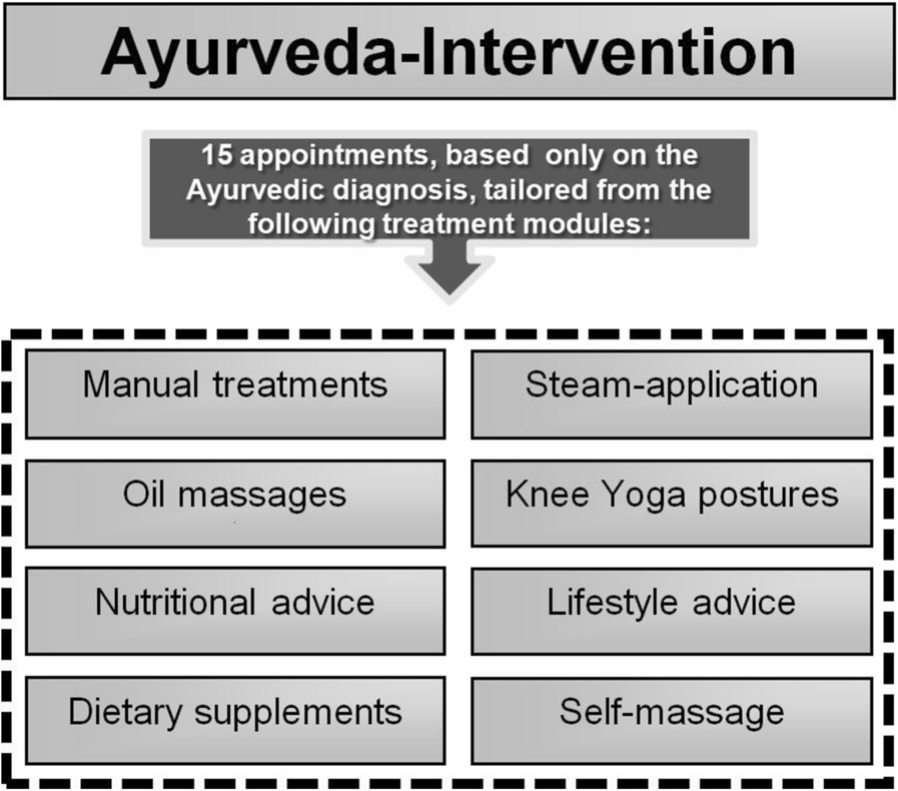
Ayurveda intervention.

Treatment includes a tailored combination of manual treatments and massages, nutritional advice, specific consideration of selected food items and nutritional supplements, general and specific lifestyle advice, yoga posture advice for the knee and daily self-applied home knee massage. Patients receive 15 treatment sessions up to a maximum of 90 minutes each (Figure 2). To ensure the highest treatment quality, the therapeutic regimes for the first 30 patients were outlined independently by four Ayurvedic medical experts and the patients received the consensus-based therapy.

#### Conventional care group

Patients randomized into the control group receive conventional standard care for osteoarthritis of the knee according to the current international guidelines [4,89,90]. Conventional standard care treatment is provided by orthopedic specialists, surgeons, physiotherapists, and occupational therapists. In Germany, OA is usually treated by orthopedic specialists or surgeons, according to treatment guidelines issued by the German Association of Orthopedics.

**Figure 3:**
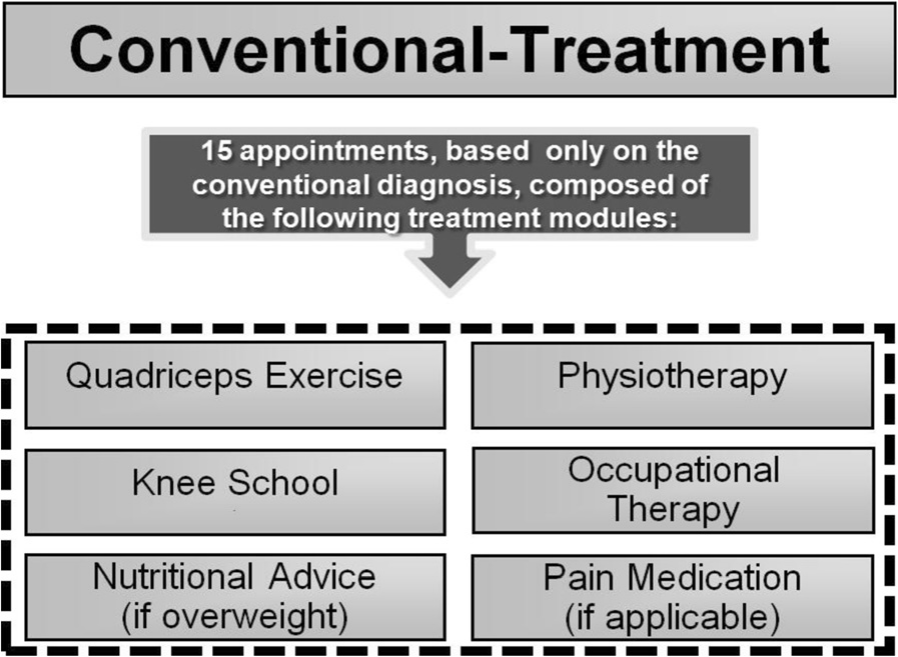
Conventional treatment.

The treatment (Figure 3) is individualized and includes quadriceps muscle strengthening exercises, local physiotherapy including manual therapy and friction massage, occupational therapy, advice for individual knee exercise (knee school), if a patient is overweight or obese dietary advice for weight loss, if necessary administration of medication according to the current guidelines. Patients receive 15 sessions with a duration of 45 minutes.

#### Both groups

In both treatment groups, a maximum of 3g paracetamol per day can be used as rescue medication. In case of intolerance or unresponsiveness to paracetamol, topical or oral NSAIDs can be used, on the advice of a study physician (for example, diclofenac-sodium ointment three times a day or oral ibuprofen up to a maximum dose of 800 mg daily or equivalent. For safety reasons, this dosage of oral NSAID was kept low.)

### Selection and qualification of providers and treatment context

In the Ayurveda group all involved medical doctors had undergone either a regular university program for Ayurveda in India (Indian experts) or at least 500 hours of academic training in Ayurveda plus at least 2 years of continuous clinical experience with Ayurveda (European experts). All other involved Ayurvedic therapists require a minimum of 2 years of continuous clinical experience in their corresponding field of expertise (manual therapies, nutritional advice, lifestyle advice, knee yoga counseling). To ensure the highest treatment quality, the therapeutic regimes for the first 30 patients were outlined independently by four Ayurvedic medical experts (all medical doctors) and the patients received the consensus-based therapy. This was assessed in an embedded diagnostic trial; the results will be presented elsewhere.

In the control group, the individual intervention is prescribed by medical doctors with a specialized degree in orthopedics, surgery, or physical medicine or by other medical doctors working in orthopedics, orthopedic surgery, or physical medicine under the direct guidance of a specialist for orthopedics or orthopedic surgery. All other involved therapists (physiotherapy, occupational therapy) require a completed training in their field and a minimum of 2 years of continuous clinical experience. The intervention takes place in hospital departments or hospital-affiliated centers for orthopedics, surgery, physical medicine, physiotherapy, or occupational therapy.

### Statistics

This study is designed to have 80% power to detect a 10-point improvement (change from baseline) on the WOMAC Index after 12 weeks between both groups (common standard deviation = 20, two-sided *t*-test *α* = 0.05). To achieve this, 64 patients per group are needed; to take dropouts into account, 75 patients per group will be included, resulting in a total of 150 patients.

The primary analysis population is the intention-to-treat population, which includes all randomized patients, who provided baseline data, regardless whether or not they adhere to the protocol or give a complete set of data.

The statistical hypotheses are:

*H*_0_: mean WOMAC Index (Ayurveda) = mean WOMAC Index (conventional)

*H*_A_: mean WOMAC Index (Ayurveda) ≠ mean WOMAC Index (conventional)

The primary outcome is a change in the score on the WOMAC Index after 12 weeks. Missing data will be multiply imputed by maximum-likelihood based regression methods. Overall, 20 complete datasets will be generated. Generalized linear mixed models will be fitted to each of these datasets, including study centers as a random effect, treatment group as a fixed factor, and the patient’s baseline WOMAC score as a linear covariate. The 20 concurring results (one for each copy of datasets) will be adequately combined. Results will be presented as adjusted WOMAC means per group with 95% confidence intervals and two-sided *P* value for the treatment group comparison.

For sensitivity analysis, this statistical model will be extended to include the patients’ and physicians’ expectations as ordinal fixed factors to the generalized linear mixed model.

Additional sensitivity analyses include the use of other computation methods, calculation of results based on the full analysis set (patients without missing WOMAC data at week 12), and the inclusion of potential confounding factors in the model in case of relevant baseline differences between the treatment groups.

A detailed statistical analysis plan will be developed before analyzing the data.

### Positioning of the trial within the efficacy-effectiveness continuum

**Figure 4:**
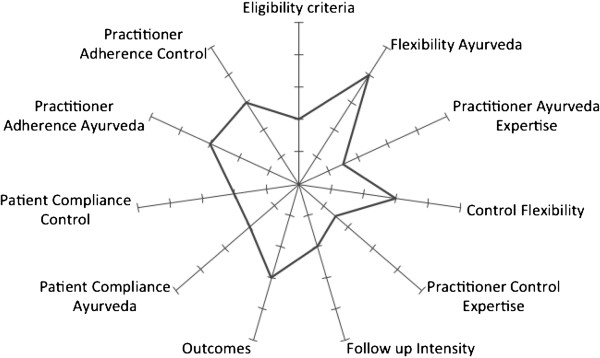
Placement of the trial in the efficacy/effectiveness continuum (efficacy is towards the center, effectiveness towards the edge).

It is essential to distinguish between ‘efficacy’ and ‘effectiveness’. ‘Efficacy’ refers to ‘the extent to which a specific intervention is beneficial under ideal conditions’ [91]. Many randomized controlled trials are efficacy trials, particularly those conducted for regulatory drug approval. They aim to produce the expected result for an intervention under carefully controlled conditions chosen to maximize the likelihood of observing an effect, if it exists. The trial population and setting of efficacy trials can differ in important ways from the clinical settings in which the interventions are likely to be used [92]. By contrast, ‘effectiveness’ is a measure of the extent to which an intervention, when deployed in the field in routine circumstances, does what it is intended to do for a specific population [91] and therefore can often be more relevant to policy evaluation and the healthcare decisions of providers and patients. The pragmatic-explanatory continuum indicator summary (PRECIS) tool [93] was used to guide the design of the trial along ten dimensions of the efficacy-effectiveness continuum. PRECIS was adapted rating the items patients’ compliance and practitioners’ adherence for both interventions separately, and deleting the item primary analyses. Seven authors (CMW, Andreas Michalsen, CK, Antonio Morandi, LK, SG, MM) independently carried out the first rating using a five-point scale (1, maximum efficacy, displayed in the center of the figure; five, maximum effectiveness, displayed at the edge of the figure [94]). Results were displayed and harmonized in a second rating. The trial is multidimensional and can be seen to lie mainly in the middle of the efficacy-effectiveness continuum (Figure 4). As shown in the figure, the flexibility of both interventions (Ayurveda and conventional care) is high and represents more the effectiveness side of the trial, whereas follow-up intensity, practitioner adherence, and patient compliance represent more the efficacy side. This represents very well the efforts that have been made during the trial-planning phase to produce a trial that allows a patient-centered individualized treatment, but at the same side tries to exclude some bias.

## Discussion

This trial compares for the first time the effectiveness of a complex Ayurvedic intervention with a complex conventional intervention in a Western medical setting. Our aim was to develop a high-quality study that compared both medical systems ‘head-to-head’ and allows a complex tailored intervention in both groups. Reviews of research studies on Ayurvedic medicine so far reveal that the majority of them are experimental, focusing on single herbs, formulations, or therapies. A minority of existing Ayurveda research is clinical, focused either on medication only or on nonpharmacologic interventions, such as yoga. Most of these trials have methodological limitations ([21,95]).

Recently, a change of this trend could be observed in a randomized controlled trial on rheumatoid arthritis, successfully demonstrating that the individualized Ayurvedic treatment approach can be incorporated in a standard randomized controlled trial and that blinding can be successful, even where more than one placebo preparation is required [96,97]. However, although the work of Furst *et al*. was of great importance for future randomized controlled trial studies on other CAM disciplines [98], in particular on whole medical systems, it did not fully test the multidimensional aspect of Ayurvedic therapy, since it included the pharmacological dimension only. Overall, Ayurveda has rarely been studied in its whole and multidimensional perspective and in the manner in which it is actually being practiced.

The main challenge is to develop novel and innovative clinical study protocols to evaluate whether Ayurveda and its logic foundations are able to generate clinical evidence in full accordance with modern research methodologies. This will eventually allow the integration of traditional medical knowledge into modern science-based medicine.

This clinical trial is the first attempt in this direction, since it makes full use of the complex and multidimensional therapeutic methodology of Ayurveda, individualized according to the patient’s needs. Nevertheless, using a trial design where neither patients nor providers or assessors can be blinded introduces bias. Expectation is discussed as one of the prominent mechanisms of the placebo effect [99]; by controlling for patients’ expectation in the statistical analyses, we try to reduce this bias. Another limitation is that the consultation duration between patients and providers differs in both intervention groups. However, this reflects the usual care setting of both medical systems. Reducing the consultation duration in the Ayurvedic group would not allow adequate treatment while reducing the consultation duration in the conventional group would introduce an artificial setting. In usual care, those patients who do not respond well to conventional treatment often become interested in complementary medicine. This could have introduced a bias towards Ayurveda. One method to reduce this bias could have been to recruit only incident cases of OA of the knee. However, this would have resulted in recruitment problems and less generalizability of the results, because patients usually seek complementary medicine treatments after they have already had the disease for some time.

The Ayurvedic treatment was developed in a consensus procedure with Indian and European Ayurveda experts. The treatment protocol had to take into account German standards of medical care and the availability of Ayurvedic interventions in Germany. Because the Ayurveda treatment was based on Ayurvedic diagnoses, a diagnostic validation study was included to train the Ayurveda study physician. To ensure high-quality personalized treatment in the conventional group, the complex conventional intervention was based on current treatment guidelines for osteoarthritis of the knee and the final treatment protocol was developed in a consensus procedure with orthopedists and physiotherapists.

In the conventional treatment group, intra-articular corticosteroid injections were not part of the treatment protocol, because in Germany many patients with osteoarthritis of the knee refuse this treatment. Furthermore, medication dosage was adapted to reduce the risk of gastrointestinal bleeding. Being aware that our approach reduces the generalizability of the results to other countries, we excluded them with the aim of reducing selection bias, to reduce the difference in the characteristics of those who are selected for the study versus those who are not.

Although the study aimed to perform a ‘head-to-head’ comparison, for ethical reasons patients in the Ayurveda group are allowed to take rescue pain medication. This was the main reason for using a statistical hypothesis to determine whether Ayurveda is superior to conventional treatment, instead of using a noninferiority or equivalence approach. In the worst case, if both groups receive similar amounts of pain medication, the main difference between them would be in the different therapies offered to the two groups. The conventional group receives 15 45-minute sessions of individualized therapy including quadriceps muscle strengthening exercises, local physiotherapy including manual therapy and friction massage, occupational therapy, advice for individual knee exercise (knee school), and, where a patient is overweight or obese, dietary advice for weight loss. The Ayurveda treatment is a tailored combination of manual treatments and massages, nutritional advice, specific consideration of selected food items and nutritional supplements, general and specific lifestyle advice, yoga posture advice for the knee and daily self-applied home knee massage, in 15 treatment sessions lasting up to a maximum of 90 minutes each.

In designing the trial, aspects of efficacy and effectiveness were discussed in depth; the resulting design is a compromise between rigor and pragmatism. We decided to apply several exclusion criteria to achieve a more homogeneous study population, for two reasons: safety and less variance in the outcomes. Although the treatment protocol for both groups allows flexibility, having only a very small number of centers trained in the diagnostic procedures and high-quality experts providing the treatment limits the degree to which these results can be generalized. Furthermore, controlling compliance and adherence represents elements of efficacy. However, this is the first ‘head-to head’ comparison in the West, where Ayurveda is mainly known as wellness intervention; it is useful to run a smaller trial in a more controlled environment before starting a large multicenter trial.

Effectiveness trials on traditional medical systems obviously have to deal with difficulties based on system-dependent aspects of nomenclature, approaches, and paradigms. Future trials have to consider this and should try to incorporate both approaches into the study designs, to allow a better comparison of the systems involved. Accordingly, future trial methodology in this field cannot merely follow typical clinical drug trial methodology (‘one treatment fits all patients’), but should use an innovative approach and take traditional aspects from a whole-medical-system perspective into account, without at the same time violating the principles of evidence-based medicine research.

For future research on Ayurveda, designs that allow more flexible interventions from a whole-systems Ayurvedic approach perspective (for example, dynamic principles, individuality and a tailored treatment approach, the concept of constitution, the milieu interior concept, and a focus on prevention and salutogenesis) should be tested.

## Trial status

The study is recruiting patients.

## Abbreviations

CAM: Complementary and alternative medicine; MRI: Magnetic resonance imaging; NSAID: Nonsteroidal anti-inflammatory drugs; OA: Osteoarthritis; PRECIS: Pragmatic-explanatory continuum indicator summary; WOMAC: Western Ontario and McMaster University Osteoarthritis Index; WHO: World Health Organization; WMS: Whole Medical System.

## Competing interests

There are no financial or nonfinancial competing interests to declare in relation to this manuscript by any of the authors.

## Authors’ contributions

CMW and CK drafted the paper and guided the group of authors through the editorial process. SR edited the section on statistics. Andreas Michalsen and MM worked substantially on all conventional aspects of the paper. Antonio Morandi, ES, SG, SH, MR, and LK took part in developing all parts related to Ayurveda and whole medical systems. All authors read and approved the final manuscript.

## References

[B1] National Institute for Health and Clinical Excellence (NICE) Osteoarthritis National Clinical Guideline for Care and Management in Adults 2008 London: Royal College of Physicians 21290638

[B2] Conaghan PG Dickson J Grant RL Care and management of osteoarthritis in adults: summary of NICE guidance BMJ 2008 336 502 503 10.1136/bmj.39490.608009.AD 18310005 PMC2258394

[B3] American College of Rheumatology Subcommittee on Osteoarthritis Guidelines Recommendations for the medical management of osteoarthritis of the hip and knee: 2000 update Arthritis Rheum 2000 43 1905 1915 11014340 10.1002/1529-0131(200009)43:9<1905::AID-ANR1>3.0.CO;2-P

[B4] Jordan KM Arden NK Doherty M Bannwarth B Bijlsma JW Dieppe P Gunther K Hauselmann H Herrero-Beaumont G Kaklamanis P Lohmander S Leeb B Lequesne M Mazieres B Martin-Mola E Pavelka K Pendleton A Punzi L Serni U Swoboda B Verbruggen G Zimmerman-Gorska I Dougados M EULAR recommendations 2003: an evidence based approach to the management of knee osteoarthritis: report of a task force of the Standing Committee for International Clinical Studies Including Therapeutic Trials (ESCISIT) Ann Rheum Dis 2003 62 1145 1155 10.1136/ard.2003.011742 14644851 PMC1754382

[B5] Denoeud L Mazieres B Payen-Champenois C Ravaud P First line treatment of knee osteoarthritis in outpatients in France: adherence to the EULAR 2000 recommendations and factors influencing adherence Ann Rheum Dis 2005 64 70 74 10.1136/ard.2003.015263 15608302 PMC1755176

[B6] Cecil RL Goldman L Cecil Textbook of Medicine 2008 Philadelphia, PA: Saunders, Elsevier

[B7] Roddy E Doherty M Guidelines for management of osteoarthritis published by the American College of Rheumatology and the European League Against Rheumatism: why are they so different? Rheum Dis Clin North Am 2003 29 717 731 10.1016/S0889-857X(03)00063-2 14603579

[B8] Tramer MR Moore RA Reynolds DJ McQuay HJ Quantitative estimation of rare adverse events which follow a biological progression: a new model applied to chronic NSAID use Pain 2000 85 169 182 10.1016/S0304-3959(99)00267-5 10692616

[B9] Vaithianathan R Hockey PM Moore TJ Bates DW Iatrogenic Effects of COX-2 inhibitors in the US population: findings from the Medical Expenditure Panel Survey Drug Saf 2009 32 335 343 10.2165/00002018-200932040-00007 19388724

[B10] Institut für Demoskopie Naturheilmittel 2010. Ergebnisse einer bevölkerungsrepräsentativen Befragung 2010 Allensbach

[B11] World Health Organization Traditional Medicine in Asia 2002 New Delhi: WHO Regional Publications

[B12] World Health Organization Traditional Medicine Strategy 2002 Geneva: WHO Regional Publications

[B13] Kessler C Michalsen A The role of whole medical systems in global medicine Forsch Komplementmed 2012 19 65 66 10.1159/000338294 22737745

[B14] Association of Ayurvedic Physicians of India (AAPI) [http://apiindia.org]

[B15] World Health Organization Benchmarks for Training in Traditional / Complementary and Alternative Medicine: Benchmarks for Training in Ayurveda 2010 Geneva: World Health Organization

[B16] Morandi A Tosto C Ayurvedic point: the Italian way to Ayurveda J Ayurveda Integr Med 2010 1 141 145 10.4103/0975-9476.65086 21836805 PMC3151385

[B17] Morandi A Tosto C Ayurveda, the paradigm for personalized medicine EPMA J 2011 2 S152 S153

[B18] Morandi A Tosto C Roberti Di Sarsina P Dalla Libera D Salutogenesis and Ayurveda: indications for public health management EPMA J 2011 2 459 465 10.1007/s13167-011-0132-8 23194327 PMC3405400

[B19] Cooper EL Ayurveda and eCAM: a closer connection Evid Based Complement Alternat Med 2008 5 121 122 10.1093/ecam/nen035 18604248 PMC2396467

[B20] Cooper EL Ayurveda is embraced by eCAM Evid Based Complement Alternat Med 2008 5 1 2 10.1093/ecam/nen016 18317542 PMC2249751

[B21] Kessler C Wirksamkeit von Ayurveda bei chronischen Erkrankungen. Systematische Analysen klinischer Ayurveda-Studien 2007 Essen: KVC Verlag

[B22] Bagla P Traditional medicine. Piercing the veil of Ayurveda Science 2011 334 1491 10.1126/science.334.6062.1491 22174227

[B23] Dash V Sharma RK Caraka Samhita: Text with English Translation and Critical Exposition based on Cakrapani Datta’s Ayurveda Dipika 2001 New Delhi: Chaukhamba Sanskrit Series Office

[B24] Board of Scholars (Translator) Astangahrdaya Samhita of Vagbhata. The book of Eight Branches of Ayurveda 1999 Delhi: Vedams

[B25] Gupta SN Stapelfeld E Praxis Ayurveda-Medizin. Kāya-cikitsā. Therapiekonzepte für innere Erkrankungen 2009 Stuttgart: Haug-Publishers

[B26] Bhat S Gupta V Srikanth N Central Council for Research in Ayurveda and Siddha (India) Feasibility of Integrating Ayurveda with Modern System of Medicine in a Tertiary Care Hospital for Management of Osteoarthritis (Knee): An Operational Study. CCRS, WHO India Country Office Collaborative Study: Technical Report 2007 New Delhi: Central Council for Research in Ayurveda and Siddha, Dept. of AYUSH, Ministry of Health & Family Welfare, Govt. of India

[B27] Shri Kanta Murthy (translator) Bhava Prakasha 1998 Varanasi, India: Chaukhamba Orientalia

[B28] Srikanatha Murthy KR Madhava Nidanam (Roga Viniscaya) of Mahavakara (A Treatise on Ayurveda) 2001 Varanasi: Chaukambha Orientala

[B29] Babu S Shetty M Yoga Ratnakara (Sanskrit Text with English Translation and Explanatory Notes) 2005 Varanasi: Chaukhamba Sanskrit Series Office

[B30] Aggarwal S Negi S Jha P Singh PK Stobdan T Pasha MA Ghosh S Agrawal A Prasher B Mukerji M EGLN1 involvement in high-altitude adaptation revealed through genetic analysis of extreme constitution types defined in Ayurveda Proc Natl Acad Sci USA 2010 107 18961 18966 10.1073/pnas.1006108107 20956315 PMC2973881

[B31] Bhushan P Kalpana J Arvind C Classification of human population based on HLA gene polymorphism and the concept of Prakriti in Ayurveda J Altern Complement Med 2005 11 349 353 10.1089/acm.2005.11.349 15865503

[B32] Ghodke Y Joshi K Patwardhan B Traditional medicine to modern pharmacogenomics: Ayurveda Prakriti type and CYP2C19 gene polymorphism associated with the metabolic variability Evid Based Complement Alternat Med 2011 2011 249528 20015960 10.1093/ecam/nep206 PMC3135904

[B33] Prasher B Negi S Aggarwal S Mandal AK Sethi TP Deshmukh SR Purohit SG Sengupta S Khanna S Mohammad F Garg G Brahmachari SK Mukerji M Whole genome expression and biochemical correlates of extreme constitutional types defined in Ayurveda J Transl Med 2008 6 48 10.1186/1479-5876-6-48 18782426 PMC2562368

[B34] Rizzo-Sierra CV Ayurvedic genomics, constitutional psychology, and endocrinology: the missing connection J Altern Complement Med 2011 17 465 468 10.1089/acm.2010.0412 21563964

[B35] Tripathi PK Patwardhan K Singh G The basic cardiovascular responses to postural changes, exercise, and cold pressor test: do they vary in accordance with the dual constitutional types of Ayurveda? Evid Based Complement Alternat Med 2011 2011 251850 20953421 10.1155/2011/251850 PMC2952295

[B36] Chandanwale ASKK Clinical evaluation of Rumalaya forte in osteoarthritis Medicine Update 2007 10 23 26

[B37] Kulkarni RR Patki PS Jog VP Gandage SG Patwardhan B Treatment of osteoarthritis with a herbomineral formulation: a double-blind, placebo-controlled, cross-over study J Ethnopharmacol 1991 33 91 95 10.1016/0378-8741(91)90167-C 1943180

[B38] Chopra A Saluja M Tillu G Venugopalan A Sarmukaddam S Raut AK Bichile L Narsimulu G Handa R Patwardhan B A randomized controlled exploratory evaluation of standardized Ayurvedic formulations in symptomatic osteoarthritis knees: a Government of India NMITLI Project Evid Based Complement Alternat Med 2011 2011 724291 20981160 10.1155/2011/724291 PMC2964493

[B39] Upadhyay L Tripathi K Clinical evaluation of the efficacy of JT 2000 (Rumalaya forte) in the management of osteoarthritis: a double-blind placebo-controlled trial Medicine Update 2004 11 31

[B40] Srivastava N Sadh R Jain B Kolhapure S Evaluation and comparative clinical efficacy and safety of Rumalaya forte in patients suffering from osteoarthritis of the knee Indian J Clin Prac 2005 16 19 30

[B41] Sontakke S Thawani V Pimpalkhute S Kabra P Babhulkar S Hingorani L Open, randomized, controlled clinica trial of *Boswellia serrata* extract as compared to valdecoxib in osteoarthritis of knee Indian J Pharmacol 2007 39 27 29 10.4103/0253-7613.30759

[B42] Singh U Kishore K Seth S Comparative clinical study of indigenous drug with ibuprofen in patients of osteoarthritis Indian J Phys Rehabil [IJPMR] 1997 8–9 29 30

[B43] Sengupta K Krishnaraju AV Vishal AA Mishra A Trimurtulu G Sarma KV Raychaudhuri SK Raychaudhuri SP Comparative efficacy and tolerability of 5-Loxin® and Aflapin® against osteoarthritis of the knee: a double blind, randomized, placebo controlled clinical study Int J Med Sci 2010 7 366 377 21060724 10.7150/ijms.7.366 PMC2974165

[B44] Rastogi S Sivaraman ST Evaluation the safety and efficacy of *Rumalaya forte:* a double-blind clinical trial Orthopaedics Today 2003 5 63 65

[B45] Nachinolcar UC Prasad SR Mitra SK *Rumalaya forte* and Reosto in osteoarthritis: a combined study Medicine Update 2007 15 27 35

[B46] Kuptniratsaikul V Thanakhumtorn S Chinswangwatanakul P Wattanamongkonsil L Thamlikitkul V Efficacy and safety of *Curcuma domestica* extracts in patients with knee osteoarthritis J Altern Complement Med 2009 15 891 897 10.1089/acm.2008.0186 19678780

[B47] Kotwani A Bapna JS Dhaon BK Efficacy of an Ayurvedic gel in osteoarthritis - a parallel randomized double blind placebo controlled study Indian J Pharmacol 1997 29 40

[B48] Kimmatkar N Thawani V Hingorani L Khiyani R Efficacy and tolerability of *Boswellia serrata* extract in treatment of osteoarthritis of knee - a randomized double blind placebo controlled trial Phytomedicine 2003 10 3 7 10.1078/094471103321648593 12622457

[B49] Khare KD Khare R Kolhapure SA Evaluation of the efficacy and safety of JT-2000* in osteoarthritis: a randomized controlled clinical trial Indian J Clin Prac 2004 14 42 47

[B50] Chopra A Lavin P Patwardhan B Chitre D A 32-week randomized, placebo-controlled clinical evaluation of RA-11, an Ayurvedic drug, on osteoarthritis of the knees J Clin Rheumatol 2004 10 236 245 10.1097/01.rhu.0000138087.47382.6d 17043520

[B51] Agarwal ND Gureja Y Khosla AC Agarwal JR Rumalaya in rheumatoid arthritis and osteoarthritis (a double-blind trial) Probe 1971 10 45 49

[B52] Raval ND Pandya TN Clinical trial of *Lepidium sativum Linn* (Chandrashura) in the management of Sandhivata (osteoarthritis) AYU (An Intern Quarterly J Res in Ayurveda) 2009 30 153 157

[B53] Taneja DK Chara GU Rumalaya in osteoarthritis Antiseptic 1975 72 527 533

[B54] Sharma A Sharma R Mohite K Management of Sandhigata vata (OA) with Shamana and Shodhana therapy - a pilot study J Res Ayurveda Siddha CCRAS 2003 24 1 10

[B55] Sen B Banerjee SK De Mazumder N Sarkar N Roychowdury A Majumdar P Studies on effect of Rumalaya in osteoarthritis Indian Med J 1980 74 151

[B56] Sandhu HS Kaler JS Rumalaya therapy in rheumatoid arthritis, osteoarthritis and periarthritis shoulder Antiseptic 1978 10 63 66

[B57] Raut P Bichile L Chopra A Patwardhan B Vaidya A Ayurvedic herbal product in osteoarthritis: 6 months comparative study with glucosamine Indian J Rheumatol 2008 3 S23

[B58] Rajoria K Kumar SS Sharma RS Sharma SN Clinical study on Laksha Guggulu, Snehana, Swedana & traction in osteoarthritis (knee joint) AYU (An Intern Quarterly J Res Ayurveda) 2010 31 80 87 10.4103/0974-8520.68192 PMC3215328 22131690

[B59] Pathak B Dwivedi KK Shukla KP Clinical evaluation of snehana. Swedana and an Ayurvedic compound drug in sandhivata *vis-a-vis* osteoarthritis JREIM 1992 11 27 34

[B60] Parmeshwar KA Tiwari S Comparative study of therapeutic efficacy of Samshodhana and Samshamna chikitsa in Sandhigata vata *vis-a-vis* osteoarthritis J Res Ayurveda and Siddha CCRAS 2003 24 21 32

[B61] Mathur HH Khurana A Mathur DD Kolhapure SA Evaluation of the efficacy and safety of JT-2000 in osteoarthrits: a comparative clinical trial Medicine Update 2004 11 31 37

[B62] Jamdar J Singh K Kuhlhalli P Chakma T Study of Ayurvedic drug (rhumayog) compared to brufen in osteoarthritis Antiseptic 2004 101 62 65

[B63] Das B Padhi MM Singh OP Deep VC Tewaris NS Panda N Clinical evaluation of nirgundi taila in the management of sandhivata Ancient Science of Life 2003 23 22 34 22557109 PMC3330953

[B64] Akhtar B Mahto RR Dave AR Shukla VD Clinical study on Sandhigata Vata w.s.r. to osteoarthritis and its management by Panchatikta Ghrita Guggulu AYU (An Intern Quarterly J Res Ayurveda) 2010 31 53 57 10.4103/0974-8520.68210 PMC3215322 22131685

[B65] Chaughan D Clinical study on the effect of RHUE cap on rheumatoid arthritis and osteoarthritis Antiseptic 2004 101 425 428

[B66] Singh BB Mishra L Aquilina N Kohlbeck F Usefulness of guggul (*Commiphora mukul*) for osteoarthritis of the knee: an experimental case study Probe 1979 18 105 11253408

[B67] Singh BB Mishra LC Vinjamury SP Aquilina N Singh VJ Shepard N The effectiveness of *Commiphora mukul* for osteoarthritis of the knee: an outcomes study Altern Ther Health Med 2003 9 74 79 12776478

[B68] Lahkar S A clinical trial of Rumalaya in osteoarthritis of knee Medicine and Surgery 1981 21 21 23

[B69] Das B Clinical trial of Rumalaya tablets and cream in osteoarthritis of knee joints Probe 1983 22 175 178

[B70] Sengupta K Alluri KV Satish AR Mishra S Golakoti T Sarma KV Dey D Raychaudhuri SP A double blind, randomized, placebo controlled study of the efficacy and safety of 5-Loxin for treatment of osteoarthritis of the knee Arthritis Res Ther 2008 10 R85 10.1186/ar2461 18667054 PMC2575633

[B71] Schneider S Schmitt G Mau H Schmitt H Sabo D Richter W Prevalence and correlates of osteoarthritis in Germany: representative data from the first national health survey Orthopäde 2005 34 782 790 15912329 10.1007/s00132-005-0812-y

[B72] Verhoef MJ Lewith G Ritenbaugh C Boon H Fleishman S Leis A Complementary and alternative medicine whole systems research: beyond identification of inadequacies of the RCT Complement Ther Med 2005 13 206 212 10.1016/j.ctim.2005.05.001 16150375

[B73] Michalsen A Klotz S Ludtke R Moebus S Spahn G Dobos GJ Effectiveness of leech therapy in osteoarthritis of the knee: a randomized, controlled trial Ann Intern Med 2003 139 724 730 10.7326/0003-4819-139-9-200311040-00006 14597456

[B74] Michalsen A Deuse U Esch T Dobos G Moebus S Effect of leeches therapy (*Hirudo medicinalis*) in painful osteoarthritis of the knee: a pilot study Ann Rheum Dis 2001 60 986 10.1136/ard.60.10.986 11589179 PMC1753399

[B75] Bliddal H Rosetzsky A Schlichting P Weidner MS Andersen LA Ibfelt HH Christensen K Jensen ON Barslev J A randomized, placebo-controlled, cross-over study of ginger extracts and ibuprofen in osteoarthritis Osteoarthr Cartil 2000 8 9 12 10.1053/joca.1999.0264 10607493

[B76] Altman RD Marcussen KC Effects of a ginger extract on knee pain in patients with osteoarthritis Arthritis Rheum 2001 44 2531 2538 10.1002/1529-0131(200111)44:11<2531::AID-ART433>3.0.CO;2-J 11710709

[B77] Conway PH Clancy C Comparative-effectiveness research - implications of the federal coordinating council’s report N Engl J Med 2009 361 330 10.1056/NEJMp0905631 19567829

[B78] Witt CM Efficacy, effectiveness, pragmatic trials - guidance on terminology and the advantages of pragmatic trials Forsch Komplementmed 2009 16 292 294 10.1159/000234904 19887807

[B79] Institute of Medicine What is comparative effectiveness research? Initial National Priorities for Comparative Effectiveness Research 2009 Washington DC: The National Academies Press 29

[B80] Altman R Asch E Bloch D Bole G Borenstein D Brandt K Christy W Cooke TD Greenwald R Hochberg M Development of criteria for the classification and reporting of osteoarthritis: classification of osteoarthritis of the knee: diagnostic and therapeutic criteria committee of the american rheumatism association Arthritis Rheum 1986 29 1039 1049 10.1002/art.1780290816 3741515

[B81] Kellgren JH Radiological assessment of osteo-arthrosis Ann Rheum Dis 1957 16 494 502 10.1136/ard.16.4.494 13498604 PMC1006995

[B82] Kellgren JH Generalized osteoarthritis and Heberden's nodes BMJ 1952 26 181 187 14896078 10.1136/bmj.1.4751.181 PMC2022370

[B83] Recht MP Resnick D Magnetic resonance imaging of articular cartilage: an overview Top Magn Reson Imaging 1998 9 328 336 9894736

[B84] Recht MP Resnick D MR imaging of articular cartilage: current status and future directions AJR Am J Roentgenol 1994 163 283 290 10.2214/ajr.163.2.8037016 8037016

[B85] Recht MP Piraino DW Paletta GA Schils JP Belhobek GH Accuracy of fat-suppressed three-dimensional spoiled gradient-echo FLASH MR imaging in the detection of patellofemoral articular cartilage abnormalities Radiology 1996 198 209 212 8539380 10.1148/radiology.198.1.8539380

[B86] Watanabe K Matsuura K Gao P Hottenbacher L Tokunaga H Nishimura K Imazu Y Reissenweber H Witt CM Traditional Japanese Kampo medicine: clinical research between modernity and traditional medicine - the state of research and methodological suggestions for the future Evid Based Complement Alternat Med 2011 2011 513842 21687585 10.1093/ecam/neq067 PMC3114407

[B87] Bellamy N Buchanan WW Goldsmith CH Campbell J Stitt LW Validation study of WOMAC: a health status instrument for measuring clinically important patient relevant outcomes to antirheumatic drug therapy in patients with osteoarthritis of the hip or knee J Rheumatol 1988 15 1833 1840 3068365

[B88] Stucki G Meier D Stucki S Michel BA Tyndall AG Dick W Theiler R Evaluation of a German version of WOMAC (Western Ontario and McMaster Universities) Arthrosis Index Z Rheumatol 1996 55 40 49 8868149

[B89] Deutsche Gesellschaft für Orthopädie und orthopädische Chirurgie und Berufsverband der Ärzte für Orthopädie Leitlinien der Orthopädie: Gonarthrose 2002 Köln: Deutscher Ärzte-Verlag

[B90] American Academy of Orthopedic Surgeons Treatment of Osteoarthritis of the Knee (Non-Arthroplasty) 2008 Rosemont, IL

[B91] Last J Spasoff RA Harris S A Dictionary of Epidemiology 2001 Oxford: Oxford University Press

[B92] Committee on Comparative Effectiveness Research Prioritization Optimizing evidence Initial National Priorities for Comparative Effectiveness Research. Institute of Medicine 2009 Washington DC: National Academies Press 31

[B93] Thorpe KE Zwarenstein M Oxman AD Treweek S Furberg CD Altman DG Tunis S Bergel E Harvey I Magid DJ Chalkidou K A pragmatic-explanatory continuum indicator summary (PRECIS): a tool to help trial designers CMAJ 2009 180 E47 E57 19372436 10.1503/cmaj.090523 PMC2679824

[B94] Witt CM Manheimer E Hammerschlag R Ludtke R Lao L Tunis SR Berman BM How well do randomized trials inform decision making: systematic review using comparative effectiveness research measures on acupuncture for back pain PLoS One 2012 7 e32399 10.1371/journal.pone.0032399 22389699 PMC3289651

[B95] Park J Ernst E Ayurvedic medicine for rheumatoid arthritis: a systematic review Semin Arthritis Rheum 2005 34 705 713 10.1016/j.semarthrit.2004.11.005 15846585

[B96] Furst DE Venkatraman MM McGann M Manohar PR Booth-LaForce C Sarin R Sekar PG Raveendran KG Mahapatra A Gopinath J Kumar PR Double-blind, randomized, controlled, pilot study comparing classic Ayurvedic medicine, methotrexate, and their combination in rheumatoid arthritis J Clin Rheumatol 2011 17 185 192 10.1097/RHU.0b013e31821c0310 21617554

[B97] Furst DE Venkatraman MM Krishna Swamy BG McGann M Booth-LaForce C Ram MP Sarin R Mahapatra A Krishna Kumar PR Well controlled, double-blind, placebo-controlled trials of classical Ayurvedic treatment are possible in rheumatoid arthritis Ann Rheum Dis 2011 70 392 393 10.1136/ard.2010.136226 20736391

[B98] Ernst E Furst DE A blueprint for placebo-controlled double-blind studies of complex, individualised interventions Focus Altern Complement Ther 2011 16 49 50 10.1111/j.2042-7166.2010.01070_7.x

[B99] Enck P Benedetti F Schedlowski M New insights into the placebo and nocebo responses Neuron 2008 59 195 206 10.1016/j.neuron.2008.06.030 18667148

